# A Comparative Proteome Analysis of *Escherichia coli* Δ*rel*A Mutant Cells

**DOI:** 10.3389/fbioe.2016.00078

**Published:** 2016-10-27

**Authors:** Sónia Carneiro, Silas Villas-Bôas, Eugénio C. Ferreira, Isabel Rocha

**Affiliations:** ^1^CEB – Centre of Biological Engineering, University of Minho, Braga, Portugal; ^2^Centre for Microbial Innovation, School of Biological Sciences, The University of Auckland, Auckland, New Zealand

**Keywords:** quantitative proteomics, iTRAQ analysis, RelA, stringent response, proteome profiling

## Abstract

The bacterial RelA-dependent stringent response exerts a strong influence over various processes. In this work, the impact of the *rel*A gene mutation in *Escherichia coli* cells was evaluated by a quantitative proteomics analysis, employing stable-isotope labeling and high-resolution mass spectrometry. Chemostat cultures of *E. coli* W3110 and Δ*rel*A mutant strains were performed at two dilution rates (0.1 and 0.2 h^−1^) to assess the influence of the *rel*A gene mutation in steady-state protein levels. A total of 121 proteins showed significant alterations in their abundance when comparing the proteome of mutant to wild-type cells. The *rel*A gene mutation induced changes on key cellular processes, including the amino acids and nucleotide biosynthesis, the lipid metabolism, transport activities, transcription and translation processes, and responses to stress. Furthermore, some of those changes were more pronounced under specific growth conditions, as the most significant differences in protein ratios were observed at one of the dilution rates. An effect of the *rel*A gene mutation in the acetate overflow was also observed, which confers interesting characteristics to this mutant strain that could be useful in the production of recombinant proteins. Overall, these results provide a valuable insight into the *E. coli* stringent response under defined steady-state conditions, suggesting that this stress response might influence multiple metabolic processes like the acetate overflow or the catabolite repression.

## Introduction

The ribosome-bound RelA enzyme is the main bacterial synthetase for guanosine tetraphosphate (ppGpp), a molecule that is at the core of the stringent response. This response is triggered by the deprivation of intracellular amino acids and is characterized by rapid alterations in transcriptional activities, whereby genes required for amino acid biosynthesis are upregulated and genes associated with cell growth are repressed. The coordination of these transcriptional shifts is now understood in some detail and many of the alterations in cellular processes have been elucidated (Chang et al., 2002; Durfee et al., 2008; Traxler et al., 2008). For example, the synthesis of ribosomal proteins was shown to be decreased during the stringent response, involving a combination of the transcriptional and translational control at the level of mRNA stability, i.e., certain ribosomal proteins are self-regulated by decreasing the translation rate of their transcripts (Chang et al., 2002).

Although the effects of *rel*A gene mutations on the global gene expression have been tested, most studies have been focused on the response of *Escherichia coli* cells under growth transitions caused by glucose–lactose diauxie (Chang et al., 2002; Traxler et al., 2008), H_2_O_2_ treatment (Chang et al., 2002), or the addition of chemical analogs that mimics amino acid starvation, such as serine hydroxymate (Durfee et al., 2008). These transient shifts give only temporary and usually growth-dependent gene expression changes in response to environmental alterations, which bias the characterization of physiological states. Continuous cultures remove these transient growth effects and provide a useful tool to address fundamental questions related to elementary microbial processes, such as growth and stress responses.

Previous work on *E. coli* chemostat cultures (Carneiro et al., 2012) has shown that the RelA activity is growth rate-dependent and differently affects the metabolism of slow- and fast-growing cultures. As such, it is relevant to understand the influence of the RelA activity in the coordination of metabolic processes under different growth conditions. It is anticipated that the *rel*A gene mutation will affect central metabolic processes, as it was previously reported (Carneiro et al., 2011), but the mechanisms by which these processes are regulated and which other entities/processes are involved is still vague. A recent study (Kanjee et al., 2012) has identified direct targeted proteins by the ppGpp alarmone, which explains to a certain extent the global physiological changes in the cellular metabolism.

In this study, a quantitative proteomic analysis, employing stable-isotope labeling and high-resolution mass spectrometry, was used to characterize *E. coli* cultures under steady-state conditions. This method has gained interest lately due to its high level of proteome coverage, accuracy in protein quantification, and high-throughput (Chong et al., 2006; Wiese et al., 2007; Schwacke et al., 2009; Karp et al., 2010; Kristjansdottir and Kron, 2010; Savitski et al., 2010). The differential labeling of proteins or proteolytic peptides by stable isotopes, followed by relative and absolute quantification by MS analysis is the basis for the determination of concentration ratios of proteins expressed in cells with different phenotypes, e.g., wild-type versus mutant. In our study, 8-plex isobaric tags for absolute and relative quantitation (iTRAQ) were used for the simultaneous relative quantification of peptides from up to eight different samples. Samples from chemostat cultivations with *E. coli* strains W3110 and Δ*rel*A mutants at two dilution rates were tested. The dilution rates of 0.1 and 0.2 h^−1^ were chosen in this study because they present distinct metabolic phenotypes as previously verified in our laboratory (Carneiro et al., 2011, 2012). In particular, at a dilution rate of 0.2 h^−1^, wild-type *E. coli* cells tend to accumulate acetate, which is characteristic of the overflow metabolism. This metabolic behavior is very relevant in recombinant bioprocesses, where the accumulation of acetate hampers protein productivity, decreasing the cost-effectiveness of these processes. Recently, it was detected that strains devoid of RelA activity can decrease the accumulation of acetate (Carneiro et al., 2012), which indicates that recombinant bioprocesses can benefit from the use of Δ*rel*A mutants strains. In order to have a comprehensive overview of the RelA activity in *E. coli* cells, we analyzed the set of up and downregulated proteins under different steady-state conditions and explored the influence of the Δ*rel*A mutation in various cellular processes.

## Materials and Methods

### Bacterial Strains and Growth Conditions

*Escherichia coli* K-12 W3110 (F-, *LAM-*, *IN*[*rrnD-rrnE*]*1*, *rph-1*) and the isogenic mutant Δ*rel*A, obtained from M. Cashel (Xiao et al., 1991), were cultivated in continuous bioreactors at 37°C, pH 7, and dissolved oxygen above 30% under fixed dilution rates: 0.1 and 0.2 h^−1^. Steady-state conditions were verified by constant optical density and glucose measurements. The pH of the culture was maintained at 7.0 by adding 2.0M NaOH or 2.0M HCl. Dissolved oxygen was maintained above 30% saturation through a cascade mode controlling the airflow and agitation speed. Chemostat cultivations were operated in a 3 L fermentor (BioFlo 3000, New Brunswick Scientific, USA) with a working volume of 1.5 L. The working volume was kept constant by withdrawing the culture broth through level control. Cultures were fed with a defined minimal medium consisting of 5 g kg^−1^ of glucose, 6 g kg^−1^ of Na_2_HPO_4_, 3 g kg^−1^ of KH_2_PO_4_, 0.5 g kg^−1^ of NaCl, 1 g kg^−1^ of NH_4_Cl, 0.015 g kg^−1^ of CaCl_2_, 0.12 g kg^−1^ of MgSO_4_⋅7H_2_O, 0.34 g kg^−1^ of thiamine, 2 mL kg^−1^ of trace-element solution [described elsewhere (Rocha and Ferreira, 2002)], and 2 mL kg^−1^ of vitamins solution [described elsewhere (Rocha and Ferreira, 2002)]. The medium was continuously fed, at least for five residence times, at a given dilution rate. The minimal medium was further supplemented with 20 mg kg^−1^ of l-isoleucine to grow the W3110 strain, while the same medium with further addition of 20 mg kg^−1^ of l-valine and 25 mg kg^−1^ of kanamycin was used to grow the Δ*rel*A mutant strain.

### Analytical Techniques

Biomass was determined by measuring culture turbidity (OD_600nm_) and cell dry weight (CDW). In order to determine CDW, 10 mL of broth were filtered by 0.2 μm filters and the retentate was dried in the microwave to a constant weight. For glucose and acetate analysis, culture broth was centrifuged at 7500 × *g* for 15 min to remove the cell debris and the supernatant was collected. The glucose concentration in the culture broth was determined by the dinitrosalicylic acid (DNS) colorimetric method (Miller, 1959). The concentrations of acetic acid in the culture broth were determined with an enzymatic test kit (Acetic acid Test kit, R-Biopharm AG, Germany).

### Protein Extraction, Digestion, and iTRAQ Labeling

Aliquots of biomass from cultures (1 mL) (in duplicate) were centrifuged and the pellets were suspended in 7 M urea, 2 M thiourea, 10 mM DTT, and 0.1% SurfactAmps X-100. They were then sonicated on ice during 30-s bursts using a Soniprep 150 probe sonicator (MSE, London, UK). Samples were again centrifuged at 16,000 × *g*, and the supernatants were harvested. Protein content was assayed by the EZQ method (Invitrogen) using 20-fold dilutions of the supernatants. Aliquots containing 50 μg of total protein underwent reduction (incubation at 40°C for 1 h) and alkylation (incubation with 50 mM iodoacetamide at pH 8.0 in the dark for 1 h) followed by quenching with further DTT. Samples were diluted 10-fold in 50 mM ammonium bicarbonate and digested by incubation with 2 μg of trypsin (Promega, Madison, WI, USA) at 37°C overnight. The resulting peptides were desalted on 10 mg Oasis SPE cartridges (Waters Corporation, MA, USA) and completely dried using a speed vacuum (Speedvac) concentrator (Thermo Savant, Holbrook, NY, USA). Dried protein digests were re-constituted with 30 μL of Dissolution Buffer from the iTRAQ Reagent Multi-Plex Kit (Applied Biosystems, Foster City, CA, USA) and labeled with 8-plex iTRAQ reagents according to the manufacturer’s instructions. Labeled material from eight different samples was then combined into one sample mixture, acidified and desalted again as above, concentrated in a Speedvac to approximately 50 μL, and finally diluted to 250 μL with 0.1% formic acid in 10% acetonitrile.

### HPLC-SCX Fractionation and Mass Spectrometry Analysis

The labeled peptides were separated from the complex mixture according to cationic charge using the strong cation-exchange (SCX) fractionation. The SCX fractionation was performed on a BioSCX II 0.3 mm × 35 mm column (Agilent Technologies, Santa Clara, CA, USA) using 10 salt-steps: 10, 20, 40, 60, 80, 100, 125, 150, 200, and 500 mM KCl. Peptide fractions were captured on a 0.3 mm × 5 mm PepMap cartridge (LC Packings, Dionex Corporation, Sunnyvale, CA, USA) before being separated on a 0.3 mm × 100 mm Zorbax 300SB-C18 column (Agilent) by high-performance liquid chromatography (HPLC). The HPLC gradient between buffer A (0.1% formic acid in water) and buffer B (0.1% formic acid in acetonitrile) was formed at 6 μL/min as follows: 10% B for the first 3 min, increasing to 35% B until 85 min, increasing to 95% B until 90 min, held at 95% until 93 min, back to 10% B at 96 min, and held there until 100 min. The LC effluent was directed into the IonSpray source of a QSTAR XL hybrid Quadrupole-Time-of-Flight mass spectrometer (Applied Biosystems) scanning from 300 to 1600 *m*/*z*. The top three most abundant multiply charged peptides were selected for MS/MS analysis (55–1600 *m*/*z*). The mass spectrometer and the HPLC system were under the control of the Analyst QS software package (Applied Biosystems).

### Protein Identification and Quantification

Peptide identification and quantification were performed using the ProteinPilot software packages (Applied Biosystems). Each MS/MS spectrum was searched against the NCBI (National Center for Biotechnology Information) protein database (http://www.ncbi.nlm.nih.gov/) containing 43657 *E. coli* K-12 sequences, and protein identification was accepted based on ProteinPilot confidence scores. The search parameters were 95% confidence for the protein identification threshold, trypsin as digest agent, iodoacetamide as cysteine alkylation, urea denaturation as special factors, and rapid ID as search effort. The detected protein threshold [Unused ProtScore (UPS), i.e., a measure of the protein confidence for a detected protein calculated from the peptide confidence for peptides from spectra that have not already been completely “used” by higher scoring winning proteins] was set to 1.3 to achieve 95% of confidence and resulted in the identification of 5511 peptides being matched to 536 distinct proteins. The spectra were also searched against the same set of protein sequences in reverse to estimate the false discovery rate (FDR), which resulted in just one protein match with an “unused score” of 1.51, giving a protein identification with a FDR below 0.2%.

Using ProteinPilot, the relative abundance of each peptide in duplicate samples from Δ*rel*A mutants cells versus wild-type cells grown at *D* = 0.1 h^−1^ was determined by dividing signature-ion peak areas at *m/z* 114 and 118 by signature-ion peak areas at *m/z* 113 and 117, respectively (corresponding to the labels chosen for each sample, i.e., 114 and 118 correspond to the tags chosen for the two replicates of mutant cells at *D* = 0.1 h^−1^ while 113 and 117 correspond to the tags chosen for the wild-type also at *D* = 0.1 h^−1^). Similarly, the level of peptides in duplicate samples from Δ*rel*A mutant cells at *D* = 0.2 h^−1^ and wild-type grown at *D* = 0.2 h^−1^ was calculated by dividing signature-ion peak areas at *m*/*z* 116 and 121 by signature-ion peak areas at *m/z* 115 and 119, respectively.

The ProGroup Algorithm (within ProteinPilot) was used to compile the results from the database search into protein groups and to report protein-based ratios of relative abundance for each condition. ProteinPilot calculates average iTRAQ ratios and estimates the *p*-value and error factor (EF) for each protein hit. Only proteins identified with a minimum of two peptides and EF values lower than 2 were considered for protein identification and relative quantification. The EF defines the quantitative 95% confidence interval (CI) of a ratio as follows:

(1) $$\text{EF}=10^{\text{95}\%\;\text{CI}},$$

where

(2) $$95\%\;\text{CI}=\text{Protein ratio}\;\left(R\right)\times \text{EF}-\frac{\text{Protein ratio}\;\left(R\right)}{\text{EF}}$$

All protein ratios were converted to log space, and the average protein expression was estimated using the following equation adapted from the ProQuant software tutorial:

(3) $$R_{w}=\frac{\sum \left(w_{i}\times x_{i}\right)}{\sum w_{i}}$$

where *w_i_* = 1/(EF of the protein), the weight for the *i*th peptide and *x_i_* = log_2_(protein ratio), the ratio for the *i*th peptide in log scale. The weighted SD*_w_* was estimated using the following equations also adapted from the ProQuant software tutorial:

(4) $${\text{SD}}_{w}=\frac{\text{SD}}{b^{0.5}}$$

where SD is the unweighted SD and $b=\frac{{\left(\sum_{i=1}^{N}w_{i}\right)}^{2}}{\sum_{i=1}^{N}w_{i}^{2}}$. For the selection of differentially expressed proteins, we considered only proteins with average expression weighted log ratios larger than 0.26 and lower than −0.32, and with an SD_w_ inferior to 1.5 times the corresponding ratio.

### Functional Enrichment and Construction of a Regulatory Subnetwork

We have also examined the enrichment of gene ontology (GO) biological processes in the differentially expressed proteins. The GOToolBox (Martin et al., 2004) was used to retrieve overrepresented GO terms related to biological processes in up and downregulated proteins. The following settings were used: annotation set – EcoCyc and EcoliWiki collaborative annotation for *E. coli* K-12; ontology – biological process; mode – all terms; reference – genome; evidence – all evidence codes.

Next, a base-network consisting in gene regulatory interactions was constructed based on transcriptional regulatory information obtained from RegulonDB (Salgado et al., 2012). This base-network was then used to construct subnetworks by filtering all interactions where at least one of the gene-coding proteins with significant alterations in their abundance between cultures (i.e., mutant and wild-type) was participating. Such an approach yielded two subnetworks corresponding to each experimental condition, i.e., dilution rates of 0.1 and 0.2 h^−1^, consisting in a total of 95 and 306 nodes, and 160 and 359 regulatory interactions, respectively.

## Results

Physiological parameters were measured in both culturing conditions (dilution rates of 0.1 and 0.2 h^−1^), for strains W3110 and Δ*rel*A showing that biomass yields were lower in the wild-type culture for the 0.2 h^−1^ dilution rate, although the differences are not significant, given the CIs. Acetate accumulation was considerably higher in the wild-type culture compared to the Δ*rel*A culture at a dilution rate of 0.2 h^−1^ (Table 1).

**Table 1:** **Growth parameters of the wild-type and Δ*rel*A mutant *E. coli* strains in aerobic glucose-limited continuous culture**.

	Wild type		Δ*rel*A mutant	
Dilution rate (h^−1^)	0.10	0.20	0.10	0.20
Biomass yield (g g^−1^)	0.44 ± 0.15	0.55 ± 0.10	0.46 ± 0.06	0.67 ± 0.30
Biomass (g L^−1^)	2.2 ± 0.3	2.7 ± 0.4	2.3 ± 0.3	3.3 ± 0.5
Glucose (g L^−1^)	0.029 ± 0.009	0.040 ± 0.003	(1)	0.023 ± 0.010
*q*_Glucose_ (g g^−1^ h^−1^)	0.23 ± 0.08	0.36 ± 0.06	0.22 ± 0.03	0.30 ± 0.13
Acetate (g L^−1^)	(1)	0.34	(1)	0.02
*q*_Acetate_ (10^−3^) (g g^−1^ h^−1^)	–	25.0 ± 3.8	–	1.1 ± 0.2

*(1) Undeterminable traces*.

Using ProteinPilot, 5511 peptides were identified and matched with the NCBI protein database from which 536 protein candidates were found. After analysis, 70 proteins show a significant increase in abundance in mutant cells when compared to wild-type cells, while 46 proteins showed a significant decrease in abundance, as listed in Table S1 in Supplementary Material. This indicates that ~22% of the identified proteins changed significantly their abundance and, hence, may play a role in RelA-dependent response.

**Figure 1:**
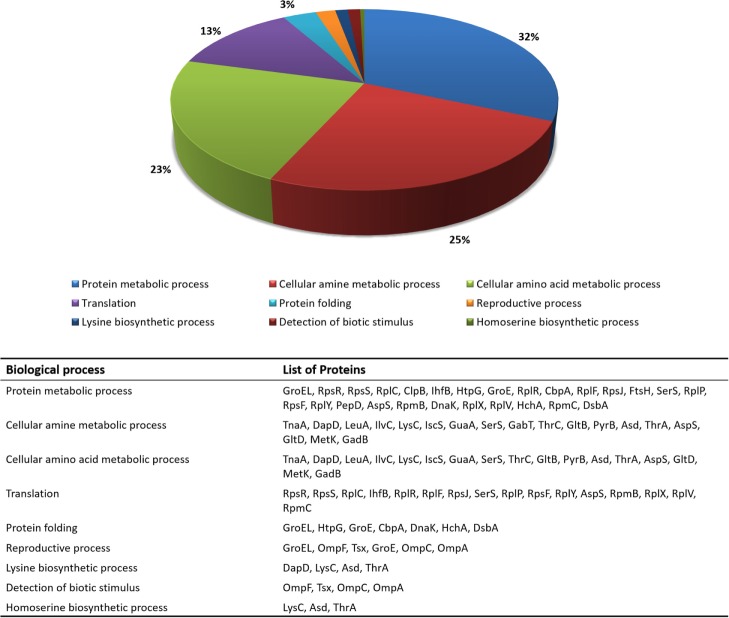
**Distribution of the most relevant biological process terms (GO) among proteins that changed in relative abundance between wild-type and Δ*rel*A mutant cells**.

The functional enrichment of the differentially expressed proteome was then examined using GO terms of biological processes. GO terms, such as translation (GO:0006412), protein metabolic process (GO:0019538), reproductive process (GO:0022414), protein folding (GO:0006457), homoserine biosynthetic process (GO:0009090), cellular amino acid biosynthetic process (GO:0008652), cellular amine metabolic process (GO:0009308), detection of biotic stimulus (GO:0009595), and lysine biosynthetic process (GO:0009085), were highlighted in the analysis. Figure 1 shows the most representative GO terms associated with biological processes for proteins with significant alterations in mutant cells.

**Figure 2:**
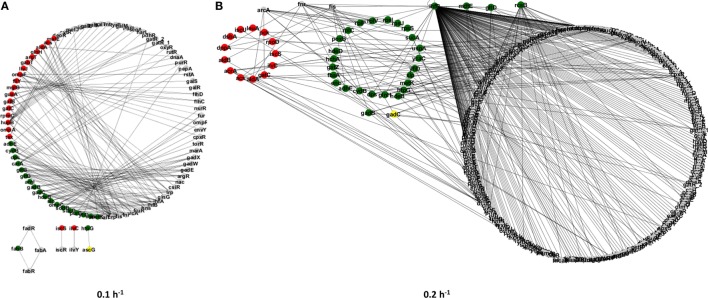
**Regulatory subnetworks representing the significant proteome changes between the *E. coli* Δ*rel*A mutant and wild-type cultures at two steady-state conditions: dilution rates of 0.1 (A) and 0.2 h^−1^ (B)**. Upregulated proteins in the mutant culture are represented by green nodes and downregulated proteins are represented by red nodes. Gray nodes represent proteins that were only detected in one of the cultures.

Next, we have constructed gene regulatory subnetworks to explore the connectivity of proteins with significant alterations in their abundance induced by the Δ*rel*A gene mutation under different steady-state conditions (see Figure 2). These subnetworks allowed us to identify cellular processes that were affected by the gene mutation and whose regulation is closely related at specific growth conditions. For instance, proteins within the same regulatory module are most likely to be involved in the RelA-dependent stringent response.

**Figure 3:**
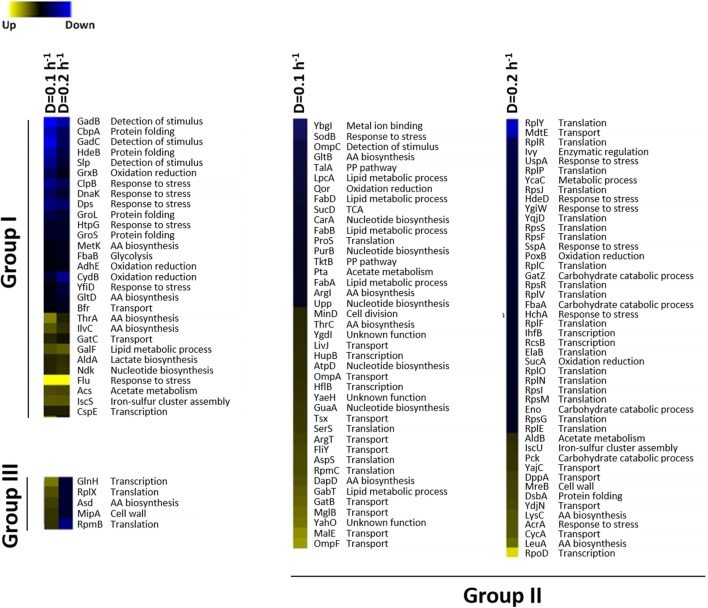
**Differential protein expression patterns in the *E. coli* Δ*rel*A mutant versus wild-type cells at two dilution rates**. Proteins included in group I were seemingly expressed in the two dilution rate conditions (0.1 or 0.2 h^−1^); group II includes proteins that were detected only in either dilution rates 0.1 or 0.2 h^−1^; and group III includes proteins for which the steady-state expression levels were opposite between dilution rate conditions. Proteins with significant upregulated expressions in the mutant cultures are colored in blue, while proteins with downregulated levels are in yellow.

In total, we have found ~121 differentially expressed proteins in each experimental condition, which involved 160 regulatory interactions for the dilution rate of 0.1 h^−1^ (Figure 2A) and almost 400 regulatory interactions for the dilution rate of 0.2 h^−1^ (Figure 2B). The increase in regulatory interactions was essentially due to the significant upregulation of regulatory proteins, such as IhfB and RcsB in the mutant culture at a higher dilution rate, which can affect a larger number of other proteins. In turn, at the dilution rate of 0.1 h^−1^ only one transcriptional regulator was found to be differentially expressed (i.e., HupB) and it regulates up to 10 genes, which resulted in a less structurally complex network. To further explore the proteome changes induced by the Δ*rel*A gene mutation, we have classified proteins into three groups (Figure 3): (I) proteins that were equally up or downregulated in cultures at both dilution rate conditions (0.1 and 0.2 h^−1^); (II) proteins that were differently expressed in only one of the conditions, i.e., either at a dilution rate of 0.1 or 0.2 h^−1^; and (III) proteins for which the expression ratios were opposite between dilution rate conditions.

### Group I

The first group consists of proteins that were consistently induced or repressed in both dilution rates and, thus, alterations in their protein levels are dependent on the gene mutation rather than on growth conditions. A total of 29 proteins were identified to respond seemingly in both growth conditions and approximately two-thirds presented upregulated levels in mutant cells. Proteins involved in the detection of *stimulus* (e.g., GadB, GadC, and Slp), response to stress (e.g., ClpB, Dps, HtpG, and YfiD), protein folding (e.g., CbpA, HdeB, DnaK, GroL, and GroS), and oxidative reduction activities (e.g., GrxB, AdhE and CydB) were upregulated in the mutant culture.

### Group II

Within the second group, a total of 41 proteins were exclusively identified in cultures run at a dilution rate of 0.1 h^−1^, among which more than half (23) were downregulated and 18 were upregulated in mutant cells. At these conditions, proteins associated with transport activities (e.g., OmpF, MalE, MglB, and GatB) were majorly downregulated in mutant cells, whereas proteins related to other metabolic processes, such as lipid biosynthesis (e.g., LpcA, FabA, FabB, and FabD) or nucleotide biosynthesis (e.g., CarA, PurB, and Upp), were upregulated. On the other hand, 46 proteins were solely identified in cultures run at a dilution rate of 0.2 h^−1^, comprising 13 proteins with downregulated expression levels in the mutant culture and 33 with upregulated levels. Proteins involved in translation activities (e.g., RplY, RplR, RplP, and RpsJ) and responses to stimulus/stress (e.g., UspA, HdeD, and YgiW) were markedly upregulated in mutant cells, while proteins related with transport (e.g., CycA and YajC) and amino acid biosynthesis (e.g., LeuA and LysC) were downregulated.

### Group III

Finally, in the last group only five proteins were identified, namely GlnH, RplX, Asd, MipA, and RpmB. Interestingly, all proteins showed downregulated expression levels in mutant cells at a dilution rate of 0.1 h^−1^ and upregulated at 0.2 h^−1^. These proteins cover cellular functions, such as translation (RplX and RpmB), transport (GlnH), and metabolic activities, such as the Asd protein that has a role in the homoserine biosynthesis and proteins with scaffolding properties in bacterial murein (MipA).

## Discussion

In this study, we sought to investigate which cellular processes are affected the most by the lack of the RelA-dependent stringent control under steady-state growth conditions. For that purpose, the proteome from continuous *E. coli* cultures were examined using iTRAQ labeling coupled with LC-MS/MS, identifying 121 differentially expressed proteins.

As it can be seen from Figure 1, a large fraction of proteins with functions in translation (GO:0006412), protein (GO:0019538) and cellular amine (GO:0009308) metabolic processes were found to have significant altered protein levels between the wild-type and Δ*rel*A mutant cultures. These results are in agreement with previous studies (Jain et al., 2006; Durfee et al., 2008; Ferullo and Lovett, 2008; Traxler et al., 2008) that associate the RelA-dependent response in *E. coli* cells with various cellular functions, such as translation and amino acid biosynthetic activities.

Although the RelA enzyme is involved in many cellular responses (Wendrich et al., 2002; Srivatsan and Wang, 2008; Li et al., 2016), it has been recognized to be central in the bacterial stringent response. Besides this enzyme, other key players have been identified, such as the SpoT bifunctional (Traxler et al., 2008; Wu and Xie, 2009; Hauryliuk et al., 2015). Though the biosynthetic activity of SpoT is less significant than that of RelA, it also contributes to the synthesis of (p)ppGpp, a signaling molecule that triggers most changes in the cell physiology during the stringent response (Xiao et al., 1991). This means that the single *rel*A mutation may not result into a completely (p)ppGpp-devoid phenotype, and as such molecular changes in *rel*A mutants cannot be attributed to the lack of a general stringent response, but as a consequence of a deficient or lower accumulation of (p)ppGpp that is essentially RelA-dependent.

In this study, an attempt is made to clarify the RelA-dependent stringent control using a systems-wide approach by monitoring proteomic profiles under different growth conditions. To better characterize the molecular changes directly associated with the cellular stringent response, mutants lacking *rel*A and *spo*T should be considered in future studies. In addition, the monitoring of (p)ppGpp levels would be relevant to investigate changes in cell physiology directly associated with this response.

The influence of the dilution rate on protein expression ratios allowed to discriminate proteins into three groups (Figure 3). Proteins in group I showed expression levels equally up- and downregulated in both growth conditions, suggesting that alterations in the protein ratios were primarily influenced by the gene mutation. Proteins related to metabolic processes, such as amino acids biosynthesis (ThrA, IlvC,), nucleotide biosynthesis (Ndk), lactate production (AldA), or acetate metabolism (Acs), were persistently downregulated in the mutant cultures, while those related to stress responses (ClpB, Dps, HtpG, and YfiD), detection of stimulus (GadB, GadC, and Slp), or protein folding (CbpA, HdeB, DnaK, GroL, and GroS) were upregulated. It seems that in the absence of the typical RelA-dependent stringent response, alternative stress responses are triggered.

**Figure 4:**
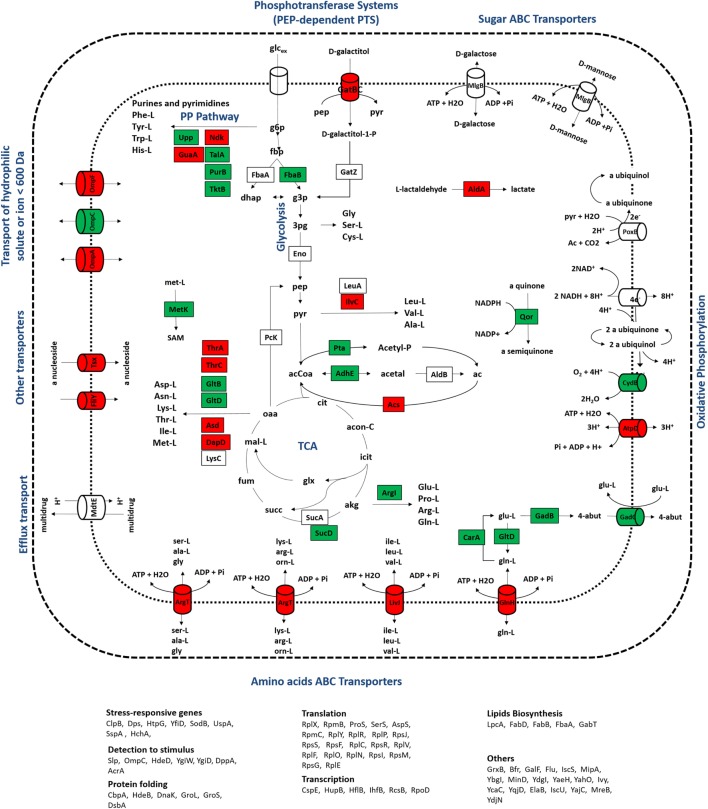
**Representation of metabolic processes associated with proteins that showed the most significant alterations in expression ratios comparing wild-type and mutant cells grown at a dilution rate of 0.1 h^−1^: downregulated (red) and upregulated (green) proteins for the mutant cultures were mapped in the metabolic map**. Uncolored proteins were represented in the map only for informative purposes.

**Figure 5:**
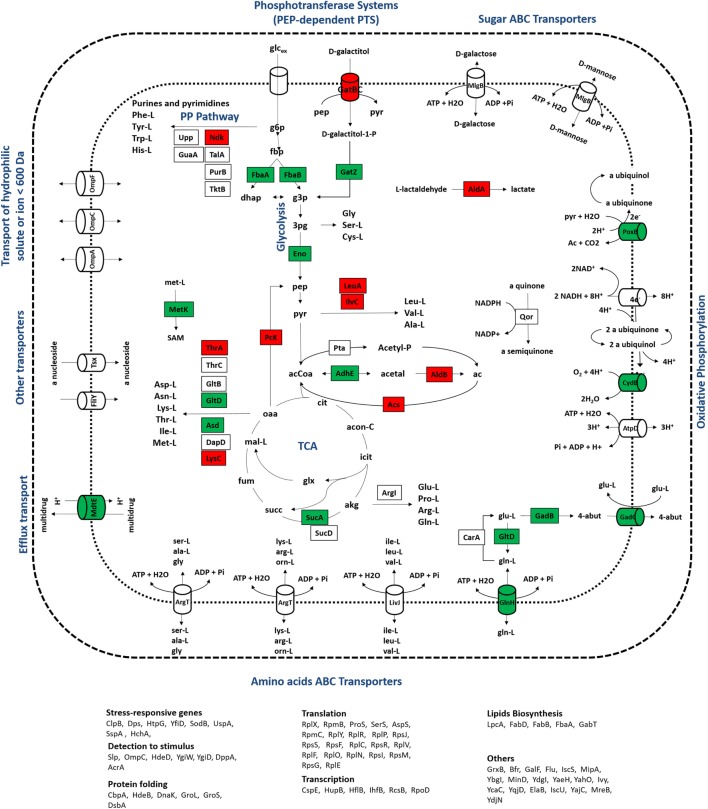
**Representation of metabolic processes associated with proteins that showed the most significant alterations in expression ratios comparing wild-type and mutant cells grown at a dilution rate of 0.2 h^−1^: downregulated (red) and upregulated (green) proteins for the mutant cultures were mapped in the metabolic map**. Uncolored proteins were represented in the map only for informative purposes.

In group II, proteins with expression levels, either significantly up- or downregulated in only one of the growth conditions, were included. Biological processes, such as alternative transport functions, appear to be negatively affected in the mutant culture at 0.1 h^−1^, revealing that unlike wild-type cells, scavenging for alternative nutrient sources was not elicited by the mutant strain. At 0.2 h^−1^, some transporters were also downregulated in mutants, but at much less extent, which may be explained by the absence of catabolite repression mechanisms that could trigger the activation of scavenging mechanisms under these conditions. This starvation-like response has been reported in other studies (Brückner and Titgemeyer, 2002; Peterson et al., 2005; Nanchen et al., 2008), associating the overexpression of alternative transporters and the activation of anaplerotic routes with this stress response, which in the absence of catabolite repression mechanisms allows for the use of alternative carbon sources. Apparently, the influence of the RelA activity on the control of catabolite repression mechanisms is significant, since visible alterations in the proteome were observed, in particular the downregulation of proteins associated with alternative catabolic activities in the mutant strain. Although the expression ratios of CRP, the catabolite regulation protein (Nanchen et al., 2008), were not significantly changed, this regulator acts as a common linker between significantly changed proteins, such as GabT, GatB, GltB, GuaA, HupB, MalE, MglB, OmpA, OmpF, SodB, SucD, and Tsx, suggesting that the regulatory activity of this module is central. Also, as illustrated in Figures 4 and 5, most of the transporters with significant proteins ratios were downregulated in the mutant culture. At these growth conditions, we can assume that the catabolite repression phenomena and, more specifically transport functions, are the most significant alterations in cellular processes derived from the Δ*rel*A gene mutation.

At a dilution rate of 0.2 h^−1^, mutant cells showed a significant upregulation of translation processes compared to the wild-type strain. The downregulation of translation functions has been described as a hallmark in the RelA-dependent stringent response (Paul et al., 2004; Durfee et al., 2008; Srivatsan and Wang, 2008). The transcription of ribosomal protein operons is inhibited by the accumulation of ppGpp, which decreases the amount of ribosomes available for protein synthesis (Traxler et al., 2006) and ultimately results in lower biomass formation. Mutants defective in the stringent response, i.e., Δ*rel*A mutants, do not accumulate high levels of ppGpp and the transcriptional control of these operons is most likely “relaxed.” For that reason, Δ*rel*A mutant cells are often said to have “relaxed” phenotypes (Herman et al., 1994). Although differences in biomass formation between wild-type and Δ*rel*A mutant cultures were not significant in this study, biomass levels were slightly higher in the Δ*rel*A mutant culture at a dilution rate of 0.2 h^−1^, which could indicate that Δ*rel*A mutants are less effective to control translation processes and, thus, growth-related processes are less stringently controlled. On the other hand, acetate production was lower in mutant cultures (having decreased from 0.34 to 0.02 g/L in the mutant culture at the dilution rate of 0.2 h^−1^), which may explain biomass levels, since acetate accumulation is known to impair cellular growth. Although it is not entirely understood how acetate overflow and the RelA-stringent control are connected, lower acetate levels in mutants suggest that the absence of the RelA-stringent control might reduce metabolic bottlenecks that ultimately lead to the accumulation of by-products, such as acetate. These results are also in agreement with metabolomics profiling analyses performed at the same conditions (Carneiro et al., 2012), where the accumulation of acetate and lactate, another metabolic by-product resulting from the metabolic overflow, was lower in mutant cultures. In this instance, we cannot state that alterations detected in protein ratios related to translation processes are caused by the gene mutation or a consequence of the reduced accumulation of acetate, but clearly the lack of the RelA activity induced profound alterations in many growth-related processes.

Also, the regulatory subnetwork illustrated in Figure 2B shows that the differentially expressed proteome at a dilution rate of 0.2 h^−1^ is structurally more complex with most proteins being upregulated in the mutant culture, including transcriptional regulators, such as the IhfB that regulates at least 8 of the differentially expressed proteins (Acs, DppA, Dps, GlnH, GltD, IhfB, SucA, and UspA). Clearly, this group comprises a set of proteins with relevant proteomic profiles, suggesting that global changes in the cellular organization of bacterial cells are elicited by the lack of the RelA activity, particularly involving cellular processes, such as translation, metabolism, and stress responses.

In the last group (III), only five proteins were detected to have significant opposite expression ratios: GlnH, RplX, RpmB, Asd, and MipA. All proteins were upregulated in the mutant culture at a dilution rate of 0.2 h^−1^ and downregulated at 0.1 h^−1^. This suggests that those proteins have cellular functions that are controlled not only by the RelA-dependent stringent response but also by growth-related processes, meaning that the influence of the RelA can be different at different dilution rates. Here, we cannot ignore the fact that by changing growth conditions different cellular processes may be elicited that intersect the cascade of regulations induced by the RelA-stringent control, which, ultimately, can influence protein expression levels in a different way (Srivatsan and Wang, 2008). For example, RplX and RpmB, two 50S subunits required for ribosome assembly, were upregulated in the mutant culture at a dilution rate of 0.2 h^−1^, but were downregulated at 0.1 h^−1^. Presumably, at a dilution rate of 0.2 h^−1^ the expression of these ribosomal proteins is influenced by the RelA-dependent control, though it is not expected that at this dilution rate the stringent response can be triggered. At a dilution rate of 0.1 h^−1^ only one translation-related protein (ProS, an aminoacyl-tRNA synthetase) was found to be upregulated in the mutant culture.

Overall, from these results, it can be hypothesized that the RelA activity can influence (directly or indirectly) crucial metabolic processes, such as the catabolite repression control and acetate overflow, that impose large alterations in the metabolic behavior of *E. coli* cells. For instance, mutant cells seem to be unable to respond to starvation-like conditions by inducing nutrient scavenging. Also, the accumulation of acetate was significantly reduced and metabolic activities, such as TCA, lipids, amino acids, and nucleotide biosynthesis, were less impaired, which confers this mutant a suitable phenotype for biosynthetic purposes, e.g., recombinant protein production. Researchers has long been searching for strains with physiological characteristics capable to increase protein productivity, and cells deficient in the stringent response have been indicated as promising recombinant hosts (Schweder et al., 1995).

A final inspection was performed by comparing the proteome profiles with the transcriptional responses of *E. coli* in other studies related to the RelA-mediated stringent control (Table 2). We sought to address gene products involved in this response by comparing with other experimental studies. Although most of the reported studies are focused on the transcriptional responses of *E. coli* cultures upon alterations in environmental conditions (e.g., diauxie conditions and serine hydroxymate or H_2_O_2_ treatments), the overlap between differentially expressed transcripts found in those studies with gene products in the present proteomics study indicates a close relationship between transcriptional responses to nutrient downshifts or starvation and the RelA-dependent control of protein expression during steady-state conditions. For instance, the downregulation of *ald*A in the mutant strain, a gene coding for the aldehyde dehydrogenase enzyme that is involved in the conversion of l-lactaldehyde into lactate, was also reported in Traxler and Chang studies (Traxler et al., 2006) upon glucose–lactose diauxie conditions. Besides gene products with metabolic functions, other transcripts were overlapped with the set of significant proteins found in this study. Transcripts associated with transcriptional processes and the translation apparatus were evidenced, most notably the rRNA-coding genes *rpl*E, *rpl*L, *rpl*X, and *rpl*Y, and the *csp*E gene, coding for the transcription anti-terminator and regulator of RNA stability. It is well-known that genes whose products are primarily involved in the translation apparatus are downregulated during the RelA-stringent control as part of the programed response to growth arrest (Barker et al., 2001; Schneider et al., 2003; Paul et al., 2004; Srivatsan and Wang, 2008). As a result, the expression levels of these genes are strongly reduced in the wild-type cells upon glucose–lactose diauxie compared to the Δ*rel*A mutant cells. This was also detected in the proteome profiles in this work, where protein levels were higher in the Δ*rel*A mutant cultures.

**Table 2:** **Gene products that are in common with microarray studies**.

Reference	Description	Protein
Durfee et al. (2008)	Time series of gene expression profiles for two serine hydroxymate-treated cultures: a wild-type *E. coli* K-12 strain, and an isogenic Δ*rel*A251 derivative defective in the stringent response	20 gene products: *argT*, *bfr*, *cspE*, *dnaK*, *elaB*, *eno*, *fbaA*, *gatZ*, *gltA*, *groL*, *groS*, *hupA*, *malE*, *ompF*, *pgk*, *ptsH*, *rplY*, *rpsF*, *sucD*, *tsx*
Traxler et al. (2006)	Changes in transcription in wild-type *E. coli* and mutants that lack RelA (ppGpp synthetase) upon glucose–lactose diauxie conditions were determined	12 gene products: *acs*, *aldA*, *bfr*, *dps*, *elaB*, *fusA*, *gatY*, *malE*, *mglB*, *rplL*, *rplR*, *rplX*
Chang et al. (2002)	Gene expression profiling of *E. coli* growth transitions caused by glucose–lactose diauxie, H_2_O_2_ treatment, and entry into the stationary phase to study the stringent control of genetic systems	26 gene products: *ahpC*, *aldA*, *atpA*, *bfr*, *cspC*, *cspE*, *dnaK*, *dps*, *fusA*, *gapA*, *glnH*, *gltB*, *glyA*, *ilvC*, *malE*, *mglB*, *ompF*, *pyrB*, *rplE*, *rplL*, *rplP*, *rplX*, *rplY*, *rpmB*, *rpsI*, *thrC*

Other transcripts involved in protein folding processes were also evidenced: *dna*K and *gro*S genes coding for molecular chaperones. The expression of these proteins may decrease after inducing stress conditions (Traxler et al., 2006; Durfee et al., 2008), but it seems that cells may not react accordingly to both stress factors. While the serine hydroxymate treatment induced a higher decrease in the expression of chaperones in mutant compared to wild-type cells (Durfee et al., 2008), diauxic conditions exerted a stronger effect on wild-type cells, decreasing even more the expression levels of chaperones (Traxler et al., 2006). The proteome profiles in this study indicate that the upregulation of these gene products was significant in the mutant cells, suggesting that to what relates with protein folding processes the behavior of these cells is closer to changes during diauxic transitions. Overall, the correlation between transcriptional changes in *E. coli* cells in response to environmental shifts and the proteome profiles detected under steady-state growth conditions of wild-type and Δ*rel*A mutant cultures suggest that the *rel*A gene mutation has a permanent effect on cellular responses.

## Conclusion

Our scope of interest in this study was not only to explore the differential proteome of the wild-type and Δ*rel*A mutant *E. coli* cultures but also in particular the effect of this single gene mutation on the steady-state cellular growth. Industrial applications of this mutant strain as a host system have been proposed to improve recombinant protein production and it is fundamental to characterize the mutant phenotype. However, the environmental and growth conditions that stimulate the RelA-dependent stringent response and how a single *rel*A gene mutation affects cellular processes at these conditions are scarcely known.

For this purpose, we investigated the proteome profiles produced by the wild-type and the isogenic Δ*rel*A mutant cultures at two fixed steady-state conditions (i.e., 0.1 and 0.2 h^−1^). This allowed to investigate the primary effects of the single gene mutation on cellular activities under carefully controlled growth conditions and to evaluate condition-specific regulatory structures that characterize the cellular behavior. As shown by the comparative proteome analysis of *E. coli* cultures at either 0.1 or 0.2 h^−1^, proteome changes were significant not only in cellular activities known to be the hallmark of the stringent response, such as translation, transcription, and amino acid biosynthetic processes, but also in stress-related and transport activities. Proteome changes induced by the gene mutation at specific growth conditions highlighted cellular processes, such as transport and translation, suggesting that these processes are central during that steady-state conditions and are under the RelA-dependent stringent control. For example, it is likely that the downregulation of transporters in mutant cultures at a dilution rate of 0.1 h^−1^ is associated with the catabolite repression phenomena, which has been reported to be shut-off during the stringent response. Changes in protein expression levels provided evidences for the involvement of the RelA activity in a wide range of processes that resulted in structurally different regulatory modules across growth conditions. These modules represent the extent of differentially expressed protein that can be interrelated by sharing common regulators and become potential hubs in the stringent control.

In conclusion, the single *rel*A gene mutation affects a large range of cellular processes even when cells are grown under steady-state conditions. The RelA-dependent response in *E. coli* cells is not only triggered by sudden environmental shifts, such as starvation conditions or diauxie transitions, but is also central to maintain many cellular activities in steady-state growth cultures. This confers to the RelA a role in processes to maintain tight control over all of the proteins needed for cell growth.

## Author Contributions

SC, EF, and SV-B designed the research. SC and IR performed the analyses. All authors interpreted the results and wrote the manuscript.

## Conflict of Interest Statement

The authors declare that the research was conducted in the absence of any commercial or financial relationships that could be construed as a potential conflict of interest.
