# Supplementary material for: A Comparative Proteome Analysis of Escherichia coli ΔrelA Mutant Cells
Source: Front Bioeng Biotechnol. 2016 Oct 27;4:78. doi: 10.3389/fbioe.2016.00078 (PMC5081369; doi:10.3389/fbioe.2016.00078)
Supplement: Supplementary file 1 [file Table_1.docx]

**Supplementary information**

**Table SI.1** - Proteins with significant changes between *E. coli* Δ*rel*A mutant *versus* wild-type cultures at two dilution rates (0.1 and 0.2 h^-1^). Protein weighted average ratios (*R_w_*) and the respective weighted standard deviation (*SD_w_*) were calculated as in Equations 3 and 4, respectively.

|  |  |  | **Log_2_ weighted** | | | |
| --- | --- | --- | --- | --- | --- | --- |
| **ProteinID** | **Protein name** | **Function** | **D=0.1 h^-1^** | | **D=0.2 h^-1^** | |
|  |  |  | ***R_w_*** | ***SD_w_*** | ***R_w_*** | ***SD_w_*** |
| ***Group I – Up-regulated in mutant cells*** | | | | | | |
| GLUTDECARBOXB-MONOMER | GadB | Glutamate decarboxylase enzyme, is part of the glutamate-dependent acid resistance system 2 (AR2) | 2,29 | 0,03 | 1,30 | 0,16 |
| XASA-MONOMER | GadC | Glutamic acid:γ-aminobutyrate antiporter, is part of the glutamate-dependent acid resistance system 2 (AR2) | 1,68 | 0,23 | 0,77 | 0,24 |
| EG12193-MONOMER | CbpA | Co-chaperone with DnaK | 1,39 | 0,18 | 0,79 | 0,15 |
| EG11399-MONOMER | HdeB | Periplasmic acid stress chaperone | 1,29 | 0,01 | 0,65 | 0,06 |
| EG11890-MONOMER | Slp | Starvation lipoprotein involded in acid resistance | 1,10 | 0,22 | 0,54 | 0,09 |
| EG10157-MONOMER | ClpB | ClpB chaperone; acts together with the DnaK chaperone system | 1,03 | 0,09 | 0,74 | 0,06 |
| EG11415-MONOMER | Dps | Dps is abundant in the stationary-phase and during normal starvation response. | 1,00 | 0,08 | 0,87 | 0,03 |
| EG10241-MONOMER | DnaK | DnaK chaperone | 0,81 | 0,02 | 0,54 | 0,04 |
| GRXB-MONOMER | GrxB | Glutaredoxin catalyzes the reduction of disulfides via reduced glutathione. | 0,73 | 0,23 | 0,35 | 0,14 |
| EG10599-MONOMER | GroL | Subunit of the chaperone protein GroEL | 0,65 | 0,09 | 0,51 | 0,02 |
| CYDB-MONOMER | CydB | Subunit of the cytochrome bd-I terminal oxidase | 0,51 | 0,21 | 1,09 | 0,09 |
| EG10461-MONOMER | HtpG | HtpG participates in folding of newly synthesized proteins under mild heat shock conditions | 0,47 | 0,13 | 0,46 | 0,04 |
| EG10600-MONOMER | groS | Subunit of the chaperone protein GroES | 0,47 | 0,07 | 0,48 | 0,04 |
| GLUSYNSMALL-MONOMER | gltD | Glutamate synthase catalyzes the conversion of L-glutamine and alpha-ketoglutarate into two molecules of L-glutamate | 0,32 | 0,06 | 0,47 | 0,10 |
| FRUCBISALD-CLASSI-MONOMER | fbaB | Fructose-bisphosphate aldolase is involved in gluconeogenesis | 0,31 | 0,00 | 0,36 | 0,08 |
| S-ADENMETSYN-MONOMER | metK | Methionine adenosyltransferase catalyzes the formation of the sulfonium compound S-adenosylmethionine | 0,31 | 0,04 | 0,39 | 0,11 |
| EG11784-MONOMER | yfiD | YfiD is assumed to be involved in stress resistance | 0,30 | 0,11 | 0,58 | 0,03 |
| ADHE-MONOMER | adhE | Acetaldehyde/alcohol dehydrogenase catalyses the interconversion between alcohols and aldehydes or ketones with the reduction of NAD^+^ to NADH | 0,29 | 0,14 | 0,32 | 0,12 |
| EG10113-MONOMER | bfr | Bacterioferritin is an iron storage protein | 0,26 | 0,04 | 0,39 | 0,06 |
| ***Group I – Down-regulated in mutant cells*** | | |  |  |  |  |
| G7080-MONOMER | flu | CP4-44 prophage is important for biofilm formation in W3110 cells grown in glucose-minimal medium | -1,91 | 0,41 | -1,84 | 0,54 |
| ASPKINIHOMOSERDEHYDROGI-MONOMER | thrA | Aspartate kinase/homoserine dehydrogenase is a bifunctional enzyme that catalyzes the first step in the biosynthesis of lysine and homoserine. | -1,02 | 0,31 | -0,42 | 0,04 |
| G7325-MONOMER | iscS | Cysteine desulfurase that catalyzes the conversion of L-cysteine into L-alanine | -0,76 | 0,02 | -0,75 | 0,03 |
| KETOLREDUCTOISOM-MONOMER | ilvC | Acetohydroxy acid isomeroreductase catalyzes a two-step process to produce L-isoleucine and L-valine | -0,74 | 0,07 | -0,50 | 0,06 |
| G7093-MONOMER | galF | GalF is a predicted subunit of a GalU/GalF protein complex involved in colanic acid building blocks biosynthesis | -0,70 | 0,36 | -0,79 | 0,47 |
| ACS-MONOMER | acs | Acetyl-CoA synthetase that catalyzes the conversion of acetate to acetyl-CoA | -0,67 | 0,03 | -0,65 | 0,00 |
| GATC-MONOMER | gatC | Subunit of the galactitol PTS permease | -0,44 | 0,11 | -0,32 | 0,06 |
| LACTALDDEHYDROG-MONOMER | aldA | Aldehyde dehydrogenase A oxidizes L-lactaldehyde to L-lactate under aerobic conditions | -0,43 | 0,01 | -0,48 | 0,17 |
| NUCLEOSIDE-DIP-KIN-MONOMER | ndk | Nucleoside diphosphate kinase converts GDP back to GTP to complete the cycle of ppGpp synthesis | -0,43 | 0,13 | -0,53 | 0,16 |
| EG12179-MONOMER | cspE | CspE belongs to the cold shock family of proteins | -0,40 | 0,11 | -0,40 | 0,04 |
| ***Group II – Up-regulated in mutant cells*** | | | | | | |
| G6379-MONOMER | ybgI | YbgI is proposed to be a hydrolase-oxidase | 0,79 | 0,50 | - | - |
| SUPEROX-DISMUTFE-MONOMER | sodB | Superoxide dismutase catalyzes the dismutation of superoxide into oxygen and hydrogen peroxide, thus providing an important antioxidant defense | 0,70 | 0,12 | - | - |
| EG10670-MONOMER | ompC | OmpC is a porin that allows ions and other hydrophilic solutes to cross the outer membrane | 0,48 | 0,11 | - | - |
| GLUSYNLARGE-MONOMER | gltB | Glutamate synthase catalyzes the production of L-glutamate from L-glutamine and alpha-ketoglutarate | 0,48 | 0,16 | - | - |
| TRANSALDOLA-MONOMER | talA | Transaldolase is an enzyme of the pentose phosphate pathway | 0,45 | 0,24 | - | - |
| G6106-MONOMER | lpcA | Sedoheptulose 7-phosphate isomerase is involved in the biosynthesis of a core component of lipopolysaccharide | 0,43 | 0,04 | - | - |
| QOR-MONOMER | qor | Quinone oxidoreductase catalyzes the reduction of quinones in a NAD(P)H-dependent reaction | 0,43 | 0,16 | - | - |
| MALONYL-COA-ACP-TRANSACYL-MONOMER | fabD | Malonyl-CoA-acyl carrier protein transacylase catalyzes one of the early reactions of fatty acid biosynthesis, the transfer of the malonyl group from malonyl-CoA to acyl carrier protein (ACP) to form malonyl-ACP | 0,39 | 0,19 | - | - |
| SUCCCOASYN-ALPHA | sucD | Subunit of the succinyl-CoA synthetase involved in TCA | 0,31 | 0,04 | - | - |
| CARBPSYN-SMALL | carA | Carbamoyl phosphate synthetase catalyzes the production of arginine and pyrimidine nucleotides. | 0,31 | 0,15 | - | - |
| FABB-MONOMER | fabB | KASI catalyzes the condensation reactions of long chain fatty acid synthesis | 0,30 | 0,04 | - | - |
| PROS-MONOMER | proS | Prolyl-tRNA synthetase is a member of the family of aminoacyl-tRNA synthetases | 0,30 | 0,04 | - | - |
| ASL-MONOMER | purB | Adenylosuccinate lyase catalyzes the *de novo* purine nucleotide biosynthesis | 0,28 | 0,17 | - | - |
| TRANSKETOII-MONOMER | tktB | TktB is responsible for the minor transketolase activity in E. coli | 0,28 | 0,05 | - | - |
| PHOSACETYLTRANS-MONOMER | pta | Phosphate acetyltransferase catalyzes the reversible conversion between acetyl-CoA and acetylphosphate, a step in the metabolism of acetate | 0,28 | 0,12 | - | - |
| FABA-MONOMER | fabA | The FabA enzyme is required for the synthesis of unsaturated fatty acids. | 0,27 | 0,06 | - | - |
| CHAINI-MONOMER | argI | Ornithine carbamoyltransferase is involved in the arginine biosynthetic pathway | 0,26 | 0,15 | - | - |
| URACIL-PRIBOSYLTRANS-MONOMER | upp | Uracil phosphoribosyltransferase is part of the pyrimidine salvage pathway | 0,26 | 0,05 | - | - |
| EG10885-MONOMER | rplY | Component of the 50S subunit of the ribosome | - | - | 1,50 | 0,16 |
| EG12240-MONOMER | mdtE | MdtE is a component of the MdtEF multidrug transporter | - | - | 1,46 | 0,41 |
| EG10879-MONOMER | rplR | Component of the 50S subunit of the ribosome | - | - | 0,59 | 0,01 |
| G6104-MONOMER | ivy | Ivy acts as a homodimer that inhibits vertebrate C-type lysozyme | - | - | 0,58 | 0,21 |
| EG11390-MONOMER | uspA | UspA is involved in response to a variety of stresses | - | - | 0,56 | 0,06 |
| EG10877-MONOMER | rplP | Component of the 50S subunit of the ribosome | - | - | 0,54 | 0,00 |
| EG11241-MONOMER | ycaC | Predicted hydrolase | - | - | 0,54 | 0,06 |
| EG10909-MONOMER | rpsJ | Component of the 30S subunit of the ribosome | - | - | 0,51 | 0,20 |
| EG11495-MONOMER | hdeD | HdeD is required for the acid resistance phenotype | - | - | 0,50 | 0,24 |
| G7574-MONOMER | ygiW | YgiW is involved in the cellular response to hydrogen peroxide and cadmium stress | - | - | 0,45 | 0,22 |
| G7612-MONOMER | yqjD | Conserved protein | - | - | 0,44 | 0,03 |
| EG10918-MONOMER | rpsS | Component of the 30S subunit of the ribosome | - | - | 0,41 | 0,13 |
| EG10905-MONOMER | rpsF | Component of the 30S subunit of the ribosome | - | - | 0,40 | 0,14 |
| EG10977-MONOMER | sspA | SspA is essential for cell survival during acid-induced stress | - | - | 0,39 | 0,11 |
| PYRUVOXID-MONOMER | poxB | Pyruvate oxidase catalyzes the oxidative decarboxylation of pyruvate into acetate and CO_2_ | - | - | 0,37 | 0,19 |
| EG10866-MONOMER | rplC | Component of the 50S subunit of the ribosome | - | - | 0,37 | 0,05 |
| G7128-MONOMER | gatZ | Tagatose-1,6-bisphosphate aldolase 2 is part of the galactitol catabolism pathway | - | - | 0,37 | 0,01 |
| EG10917-MONOMER | rpsR | Component of the 30S subunit of the ribosome | - | - | 0,37 | 0,01 |
| EG10882-MONOMER | rplV | Component of the 50S subunit of the ribosome | - | - | 0,35 | 0,01 |
| FRUCTBISALD-CLASSII-MONOMER | fbaA | Fructose 1,6-bisphosphate aldolase catalyzes the reversible cleavage of fructose 1,6-bisphosphate into glyceraldehyde 3-phosphate and dihydroxyacetone phosphate | - | - | 0,35 | 0,09 |
| G7055-MONOMER | hchA | Hsp31 is a chaperone | - | - | 0,34 | 0,16 |
| EG10869-MONOMER | rplF | Component of the 50S subunit of the ribosome | - | - | 0,32 | 0,06 |
| PD00348 | ihfB | Global regulatory protein that helps to maintain DNA architecture | - | - | 0,32 | 0,02 |
| RCSB-MONOMER | rcsB | RcsB protein is involved in the regulation of the synthesis of colanic acid capsule, cell division, periplasmic proteins and motility | - | - | 0,31 | 0,11 |
| G7173E-MONOMER | elaB | Conserved protein | - | - | 0,30 | 0,06 |
| E1O-MONOMER | sucA | 2-Oxoglutarate dehydrogenase is involved in TCA | - | - | 0,30 | 0,09 |
| EG10876-MONOMER | rplO | Component of the 50S subunit of the ribosome | - | - | 0,28 | 0,02 |
| EG10875-MONOMER | rplN | Component of the 50S subunit of the ribosome | - | - | 0,28 | 0,09 |
| EG10908-MONOMER | rpsI | Component of the 30S subunit of the ribosome | - | - | 0,27 | 0,01 |
| EG10912-MONOMER | rpsM | Component of the 30S subunit of the ribosome | - | - | 0,27 | 0,09 |
| ENOLASE-MONOMER | eno | Enolase catalyzes the interconversion of 2-phosphoglycerate and phosphoenolpyruvate in glycolysis | - | - | 0,26 | 0,02 |
| EG10906-MONOMER | rpsG | Component of the 30S subunit of the ribosome. | - | - | 0,26 | 0,09 |
| EG10868-MONOMER | rplE | Component of the 50S subunit of the ribosome | - | - | 0,26 | 0,14 |
| ***Group II – Down-regulated in mutant cells*** | | | | | | |
| EG10597-MONOMER | minD | MinD is part of the MinCD complex that inhibits the septum formation | -0,32 | 0,05 | - | - |
| THRESYN-MONOMER | thrC | Threonine synthase carries out the final step in the biosynthesis of L-threonine | -0,33 | 0,08 | - | - |
| G7453-MONOMER | ygdI | Putative lipoprotein | -0,34 | 0,11 | - | - |
| LIVJ-MONOMER | livJ | Component of the branched chain amino acids ABC transporter | -0,35 | 0,19 | - | - |
| EG10467-MONOMER | hupB | HU is a regulatory protein involved in responses to environmental changes like changes in osmolarity, acid stress, SOS induction and anaerobiosis | -0,36 | 0,07 | - | - |
| ATPD-MONOMER | atpD | ATP synthase catalyzes the synthesis of ATP under aerobic cell growth. | -0,37 | 0,14 | - | - |
| EG10669-MONOMER | ompA | OmpA is a nonspecific diffusion channel allowing small solutes to cross the outer membrane | -0,37 | 0,01 | - | - |
| EG11506-MONOMER | hflB | ATP-dependent zinc metalloprotease FtsH is involved in the degradation of aberrant membrane and cytoplasmic proteins | -0,39 | 0,13 | - | - |
| EG12336-MONOMER | yaeH | Conserved protein | -0,40 | 0,22 | - | - |
| GMP-SYN-MONOMER | guaA | GMP synthetase catalyzes the glutamine- or ammonia-dependent synthesis of GMP from XMP | -0,43 | 0,27 | - | - |
| EG11035-MONOMER | tsx | Tsx is an outer membrane porin responsible for the uptake of (deoxy)nucleosides | -0,43 | 0,05 | - | - |
| SERS-MONOMER | serS | Seryl-tRNA synthetase is a member of the family of aminoacyl-tRNA synthetases | -0,46 | 0,07 | - | - |
| ARGT-MONOMER | argT | Component of the lysine/arginine/ornithine ABC transporter | -0,52 | 0,13 | - | - |
| G7039-MONOMER | fliY | FliY participates in the transport of amino acid-like compounds | -0,55 | 0,28 | - | - |
| ASPS-MONOMER | aspS | Aspartyl-tRNA synthetase is a member of the family of aminoacyl-tRNA synthetases | -0,59 | 0,38 | - | - |
| EG10887-MONOMER | rpmC | Component of the 50S subunit of the ribosome | -0,60 | 0,29 | - | - |
| MONOMER0-2001 | dapD | Tetrahydrodipicolinate succinylase is involved in lysine biosynthetic pathway | -0,64 | 0,03 | - | - |
| GABATRANSAM-MONOMER | gabT | 4-Aminobutyrate aminotransferase is involved in the 4-aminobutyrate degradation pathway | -0,73 | 0,15 | - | - |
| GATB-MONOMER | gatB | Subunit of the galactitol PTS permease | -0,79 | 0,32 | - | - |
| MGLB-MONOMER | mglB | Periplasmic binding component of the galactose ABC transporter | -0,86 | 0,01 | - | - |
| G6194-MONOMER | yahO | Predicted protein | -0,91 | 0,04 | - | - |
| MALE-MONOMER | malE | Periplasmic substrate-binding component of the maltose ABC transporter | -1,12 | 0,05 | - | - |
| EG10671-MONOMER | ompF | Outer membrane porin F that allows the passage of solutes such as sugars, ions, and amino acids which are less than 600 daltons | -1,22 | 0,05 | - | - |
| ALDDEHYDROGB-MONOMER | aldB | Acetaldehyde dehydrogenase catalyses the production of acetate from acetaldehyde | - | - | -0,34 | 0,02 |
| G7324-MONOMER | iscU | Scaffold protein involved in iron-sulfur cluster assembly | - | - | -0,39 | 0,14 |
| PEPCARBOXYKIN-MONOMER | pck | Phosphoenolpyruvate carboxykinase catalyses the gluconeogenic production of phosphoenolpyruvate from oxaloacetate | - | - | -0,41 | 0,07 |
| EG11096-MONOMER | yajC | Component of the Sec protein secretion pathway | - | - | -0,41 | 0,08 |
| DPPA-MONOMER | dppA | Periplasmic binding component of the dipeptide ABC transporter | - | - | -0,49 | 0,13 |
| EG10608-MONOMER | mreB | Subunit of the peptidoglycan synthesis/chromosome segregation-directing complex | - | - | -0,51 | 0,05 |
| DISULFOXRED-MONOMER | dsbA | Disulfide oxidoreductase capable of oxidizing proteins very rapidly | - | - | -0,52 | 0,03 |
| G6934-MONOMER | ydjN | Predicted transporter | - | - | -0,57 | 0,38 |
| ASPKINIII-MONOMER | lysC | Aspartokinase III is one of three aspartokinase activities catalyzing the biosynthesis of lysine and homoserine | - | - | -0,64 | 0,35 |
| EG11703-MONOMER | acrA | Periplasmic lipoprotein component of the AcrAB-TolC multidrug efflux pump | - | - | -0,68 | 0,07 |
| CYCA-MONOMER | cycA | Transporter involved in the uptake of glycine, serine and alanine | - | - | -0,69 | 0,40 |
| 2-ISOPROPYLMALATESYN-MONOMER | leuA | 2-Isopropylmalate synthase carries out the first step in leucine biosynthesis | - | - | -0,85 | 0,07 |
| RPOD-MONOMER | rpoD | Sigma 70 is the primary sigma factor during exponential growth | - | - | -1,72 | 0,19 |
| ***Group III - Opposite profiles*** | | | | | | |
| GLNH-MONOMER | glnH | Component of the glutamine ABC transporter | -0,93 | 0,04 | 0,40 | 0,12 |
| EG10884-MONOMER | rplX | Component of the 50S subunit of the ribosome | -0,63 | 0,01 | 0,38 | 0,07 |
| ASP-SEMIALDEHYDE-DEHYDROGENASE-MONOMER | asd | Aspartate semialdehyde dehydrogenase carries out the middle step in the pathway of homoserine biosynthesis | -0,49 | 0,05 | 0,37 | 0,11 |
| G6968-MONOMER | mipA | Scaffolding protein that interacts with murein polymerase and murein hydrolase | -0,41 | 0,07 | 0,36 | 0,12 |
| EG10886-MONOMER | rpmB | Component of the 50S subunit of the ribosome | -0,40 | 0,03 | 0,97 | 0,05 |
